# Retraction: The variation of nasal microbiota caused by low levels of gaseous ammonia exposure in growing pigs

**DOI:** 10.3389/fmicb.2025.1697759

**Published:** 2025-09-08

**Authors:** Frontiers Editorial Office

Following publication, concerns were raised regarding the scientific validity of the article. An investigation was conducted in accordance with Frontiers' policies. The authors failed to provide a satisfactory explanation and as a result, the conclusions of the article have been deemed unreliable and the article has been retracted.

This retraction was approved by the Chief Editors of Frontiers in Microbiology and the Chief Executive Editor of Frontiers. The authors did not agree to this retraction.

